# Compatibility between Humans and Their Dogs: Benefits for Both

**DOI:** 10.3390/ani9090674

**Published:** 2019-09-12

**Authors:** Mónica Teresa González-Ramírez

**Affiliations:** School of Psychology, Facultad de Psicología, Autonomous University of Nuevo León, Nuevo León C.P. 64455, Mexico; monygzz77@yahoo.com; Tel.: +52-81-833-382-33

**Keywords:** human-animal interaction, compatibility, pet effect, stress, happiness, C-BARQ, dog behavior

## Abstract

**Simple Summary:**

Human-animal interaction and its benefits have been a topic of interest in recent decades, but how this interaction can benefit both species remains unclear. In the case of companion animals, the focus of many studies is on human-dog interaction. When they live together, humans and dogs share activities, and when they share an enjoyment of daily activities such as walking and interacting with others, they are considered compatible in their activity preferences. Individuals who are more compatible with their dogs also report having a better relationship with them, which could explain some of the benefits of human-dog interaction. In this study, ninety people with low compatibility in activity preferences were compared to 110 people who were compatible with their dogs. The findings show that the people who were more compatible with their dogs reported higher happiness levels and lower stress scores, a stable dog-feeding routine, and more frequent daily walks and playing sessions, in addition to a lower frequency of aggressive and fearful behaviors and higher trainability scores in their dogs. In conclusion, compatibility in activity preferences helps explain the benefits of the human-animal interaction.

**Abstract:**

Compatibility in activity preferences refers to the shared enjoyment of daily activities, such as walking and interacting with others, and it is an indicator of the behavioral dimension of compatibility, which mainly refers to exercise and play. It has been found that individuals who are more compatible with their dogs have a better relationship with them, which can explain some of the benefits of human-dog interaction. However, research to explain how and why human-animal relationships are potentially therapeutic is still needed. The objective of this quantitative study was to compare the benefits of human-dog interaction for both humans and dogs between people who were and were not compatible with their dogs. Ninety people with scores of 50% or less on the compatibility index and 110 people with 100% compatibility participated in the study. The groups were compared using the Mann-Whitney U test. The people in the group with greater compatibility reported more subjective happiness and less perceived stress, a stable dog-feeding routine, and more frequent daily walks and playing sessions; additionally, for their dogs, they reported a lower frequency of aggressive and fearful behaviors and higher trainability scores. In conclusion, compatibility in activity preferences helps explain the benefits of human–animal interaction.

## 1. Introduction

Companion animals are animals that live with humans and do not have an obvious function [1]. Although companion animals have no apparent function, research shows that both humans and animals benefit from this coexistence.

Adequate coexistence between the two species is facilitated when the individuals are compatible. Compatibility is the fit between a companion animal and its owner in regard to physical, behavioral and psychological dimensions [2].

For all three dimensions, the companion animal and owner have the things that they require of, as well as the things that they are able to contribute to, the relationship. The match between these requirements and contributions represents the compatibility of particular companion animal and owner combinations [2]. The basic physical requirements are water, food, shelter and health care, and most companion animals are dependent on their owner to provide these requirements. The behavioral requirements include exercise and playful interaction. Physical and behavioral requirements are relatively easily identified. An example of the psychological component would be the amount of affection that a person desires from an animal compared with the amount that the pet is prepared to offer [3].

In its behavioral dimension, compatibility has been related to lower aggressive behavior, trainability [4] and the human-dog relationship [5].

Human-dog interaction has become popular as a line of research documenting health benefits for humans. For example, data from the National Health and Nutrition Examination Survey of a representative sample of people living alone in the United States (*n* = 2474) showed that dog and cat owners had better physical health than nonowners or owners of other types of companion animals [6].

There are review studies on the subject, such as those of Amiot et al. [1] and Wells [7]. In the former study, the authors indicate that epidemiological and longitudinal studies have revealed positive associations between the presence of companion animals and human physical well-being, revealing that people who lived with companion animals had fewer doctor visits than similar people without companion animals [1].

In latter study, Wells [7] reviews the research on the relationship between the physical and psychological health of humans and companion animals, focusing on well-being and quality of life. The review shows that people exposed to a psychological or physical stressor had a lower heart rate and blood pressure in the presence of companion animals—dogs or cats—than in the presence of a friend or spouse, leading to the conclusion that in this context, companion animals function as a distraction from the stressful situation. Another study reported that in a population with hypertension, dog owners had a lower risk of fatal cardiovascular events compared to nonowners [8].

However, other studies have found opposite results. For example, the review by Wells [7] shows that there are inconsistencies and methodological limitations among studies, which leads to the conclusion that further research in areas such as depression and the benefits for people with cardiovascular diseases is needed.

Empirical evidence indicates that relationships between humans and animals benefit not only human health but also the health of the animals [1]. For example, in military dogs, time spent with their handlers is associated with a decrease in behaviors related to chronic stress in dogs [9].

Thus, both the significant and nonsignificant results of research should be considered to gain a complete picture of the relationships between humans and animals and to understand when and for whom these relationships are beneficial [10]. However, evidence and a unified theoretical framework that explains how and why human-animal relationships are potentially therapeutic are still lacking in the discipline [11].

Companion animals confer some benefits for human health, but it is not known exactly how. For example, in the previously described longitudinal study with a representative sample from the United States, it was reported that there is no increase in physical activity associated with the active care of companion animals [6], whereas another longitudinal study with a small sample (*n* = 17) found that 8 months after acquiring a dog, humans had increased their physical activity [12]. These contradictions support the need to investigate other variables that explain the benefits of the human–animal relationship and to not attribute the benefits simply owning a companion animal.

To clarify the concepts used in this research, it is necessary to explain that a relationship is dyadic and that a bond refers to the characteristics of an individual, that is, the bond of an individual with another individual [13]; thus, it can be said that a human has an emotional bond with his dog, regardless of how the animal perceives the relationship [14]. In this research, I analyze details about the human-animal relationship, specifically the way in which humans and their dogs interact: human-animal interaction.

It is possible that the interest in understanding the human-companion animal relationship has been focused more on the benefits for humans. For example, in a previous study performing structural equation modeling, it was found that the number of stressful situations faced recently and the human–dog relationship were predictors of perceived stress and that stress was a predictor of subjective happiness in humans. That study concluded that the quality of the human-dog relationship is a mediator of the benefits of human-animal interaction and that the quality of the relationship is determined by shared activities [15].

There are different ways to define stress; in this study, perceived or psychological stress is defined as a particular relationship between the individual and the environment in which the environment is appraised by the individual as threatening or as overwhelming his/her resources and in which the environment threatens his/her well-being [16].

It has been documented that humans who spend more time with their dogs in daily activities or playing at home report not only better relationships with their dogs but also lower perceived stress [15]. Thus, it is hypothesized that individuals whose activity preferences are more compatible with those of their dogs will also experience lower stress levels and possibly other benefits. Activity preferences refer to the shared enjoyment of daily activities such as walking and interacting with others, and they are an indicator of the behavioral dimension of compatibility [5], which mainly refers to exercise and play [3].

Based on the above, the objective of this study is to compare the benefits of human–dog interaction for both humans and dogs between people who share and do not share activity preferences with their dogs. As indicators of benefits, for the humans, I considered the number of doctor visits in the last year, subjective happiness and perceived stress; for the dogs, a stable feeding routine and the frequency of walks and playing sessions were measured; and also the Canine Behavioral Assessment and Research Questionnaire (C-BARQ) [17] was used to measure behaviors such as anxiety, aggressiveness and trainability.

## 2. Materials and Methods

### 2.1. Participants

Residents of Mexico who had dogs, which was the only inclusion criterion, were invited to participate in the study. A survey was completed online through SurveyMonkey.com, and the survey link was disseminated through social networks (Facebook). No person was directly contacted; using the snowball method, in which each person is asked to invite another person to respond, the survey link was posted on the author’s wall on Facebook, and contacts were asked to share it.

Snowball sampling is a useful methodology in research. In particular, in studies in which the respondents are few in number, virtual snowball sampling facilitates access to a specific population, reducing the costs and time of research. In addition, the utilization of Facebook to contact individuals is a useful means of expanding the sample size and minimizing some barriers associated with other online techniques of data collection [18].

Surveys in which the last question was not answered were discarded. All data were treated confidentially. The survey was open for a two-month period.

A total of 280 people completed the survey. Individuals with compatibility scale scores (described below) indicating 50% compatibility or less composed group 1 (*n* = 90), while those with compatibility scores equal to 100% compatibility composed group 2 (*n* = 110); the final sample included 200 participants. The characteristics of the participants and their dogs are presented in the Results section.

### 2.2. Instruments

#### 2.2.1. Compatibility of Owner-Dog Preferences

The compatibility of owner-dog preferences was evaluated with the Dog-Owner Compatibility Index of Activity Preferences [5], which consists of 12 items with 4 Likert-type response options ranging from totally disagree to totally agree (0 = totally disagree; 1 = disagree; 2 = agree; 3 = totally agree). Six items evaluate the owner’s preferences for specific activities (e.g., walking, visiting new places), and 6 evaluate the dog’s preferences for the same kinds of activities. The index is calculated as follows: First, the presence of compatibility in each of the 6 situations is determined; the owner and dog are seen as compatible if they agree (score 2 or 3 for both or score 0 or 1 for both) or disagree regarding the enjoyment of an activity; therefore, if both score the same number or similar they are still rated as compatible. Next, the number of matching responses is divided by 6, and the result is multiplied by 100. The authors who developed the scale reported a Cronbach’s alpha of 0.82; with the data from the present study, Cronbach’s alpha was 0.85.

#### 2.2.2. Indicators of Benefits for Humans

For happiness, the version of the Subjective Happiness Scale (SHS) [19] validated for the Mexican population [20] was used. The items on this scale are answered with 4 Likert-type response options. The scale shows adequate internal consistency, with alphas of 0.77 in the validation for Mexico and 0.76 in the present study.

For perceived stress, the version of the Perceived Stress Scale (PSS) [21] validated for Mexico [22] was used. This instrument contains 14 items answered on a Likert-type scale ranging from 0 = never to 4 = very often; 7 items are reverse scored. The Mexican version showed an alpha of 0.83 [22]; in the present study, the alpha was 0.86.

For the number of doctor visits in the last year, an open question was included: In the last year, how many times did you see a doctor for any illness or discomfort?

#### 2.2.3. Indicators of Benefits for Dog

For questions regarding the dog, the participants who had more than one dog were asked to answer the question thinking of only one dog. The indicators of benefits for dogs were measured with the following questions:

On the routine with the dog: How often do you play with your dog (with response options ranging from 1 = sometimes to 5 = several times a day)? Do you walk your dog at least once a day (with response options ranging from 1 = strongly disagree to 4 = strongly agree)? Does your dog eat at fixed times (with response options ranging from 1 = never to 4 = always)? Is the food bowl available to the dog all day long to eat when it is hungry (with response options ranging from 1 = never to 4 = always)?

In addition, the version of Canine Behavioral Assessment and Research Questionnaire (C-BARQ©) [17] validated for Mexico [4] was used. This instrument consists of 14 subscales assessing concrete and observable behaviors, and items are answered using a 5-point Likert-type scale. The scale evaluates the intensity of behaviors of aggression, fear and excitability and the frequency of other behaviors (zero indicates the absence of the behavior, and 4 indicates the highest intensity or frequency). The subscales of the C-BARQ are as follows:

Stranger-directed aggression: The dog displays aggressive or threatening behaviors toward strangers who approach or invade his/her personal space or that of the owner or his/her territory (her/his home).

Owner-directed aggression: The dog displays aggressive or threatening behaviors toward the owner or family members when scolded, punished, stared at or approached while eating or when he/she has an object in his/her possession.

Dog-directed aggression: The dog displays aggressive or threatening behavior when approached directly by an unfamiliar dog.

Dog rivalry: The dog displays aggressive or threatening behavior toward other familiar dogs living in the same home.

Stranger-directed fear: The dog is cautious or afraid when approached directly by a stranger.

Nonsocial fear: The dog is cautious or afraid of sudden or loud noises, heavy traffic or unfamiliar objects or situations.

Dog-directed fear: The dog is cautious or afraid when directly approached by an unknown dog.

Touch sensitivity: The dog is cautious or afraid of potentially painful or uncomfortable procedures, including bathing, brushing, claw clipping and veterinary checks.

Separation-related behavior: The dog vocalizes and/or destroys things when separated from the owner; the behavior is often accompanied or preceded by behaviors and signs of anxiety that include restlessness, loss of appetite, trembling or excessive salivation.

Attachment or attention-seeking behavior: The dog maintains close proximity to the owner or other family members, requests affection or attention and shows signs of agitation when the owner gives attention to others.

Trainability: The dog shows a willingness to attend to and obey simple instructions from the owner. It is not easily distracted, tends to learn fast, responds positively to correction, and fetches objects.

Chasing: The dog chases cats, birds and/or small animals when given the chance.

Excitability: The dog overreacts to potentially exciting events such as walks or car trips, the doorbell, the arrival of visitors, or the owner’s arrival home, and it has difficulty calming down after those events.

Energy level: The dog is full of energy, always moving or playing.

### 2.3. Statistical Analysis

The data were analyzed using descriptive statistics. IBM ^®^SPSS ^®^ Statistics 20 software; SPSS Inc., Chicago, Illinois, IL, USA) was used for the statistical analyses. To evaluate the differences between groups, the Mann–Whitney U test was used for continuous variables, and the chi-squared test was used for categorical variables. Nonparametric tests were used because the scores did not fit a normal distribution when evaluated using the Kolmogorov-Smirnov test (*p* < 0.05).

## 3. Results

### 3.1. Characteristics of the Participants and Their Dogs

The groups were equivalent in terms of most of the characteristics (Table 1 and Table 2). In addition, using the chi-square test (*p* > 0.05), no association between sterilization status or dog sex was found within each group. The dogs are not described by breed due to the large variety; instead, they are described according to size (Table 2).

### 3.2. Benefits for Humans

The results of the comparison of group 1, composed of individuals having compatibility scores of 50% or less (*n* = 90), and group 2, composed of individuals having 100% compatibility with their dogs in activity preferences (*n* = 110), are reported in this section. Table 3 shows the indicators of benefits for humans; people with 100% compatibility with their dogs reported higher subjective happiness scores and lower perceived stress scores, as well as a lower mean number of doctor visits in the last year, although the latter difference was not significant.

Within each group, with regard to happiness, stress and the number of doctor visits in the last year, no differences between sex of the dog or between its sterilization status were found (*p* > 0.05).

### 3.3. Benefits for Dogs

Questions about the routine with the dog were considered indicators of the benefits of the interaction for the dog, although the routine could also be beneficial for the human by representing habits and physical exercise. Table 4 shows significant differences in these indicators. The group whose members reported being more compatible with their dog had better habits, reflected by the greater frequency of playing with and walking their dog as well as a stable feeding routine. A total of 76% of the people in this group reported that their dog always eats on a fixed schedule, while only 47% of the those in the group with less compatibility reported the same. Regarding the frequency with which the participants played with their dog, 43% of those in the compatible group reported playing several times a day with their dog, and 50% reported playing with their dog at least once a day. In contrast, among those in the group with lower compatibility, 31% reported playing several times a day, and 37% reported playing at least once a day. When asked if they walk their dog at least once a day, 71% of the people with greater compatibility agreed or strongly agreed, while only 40% of those who reported lower compatibility gave the same answers.

Within each group, with regard to the variables reported in Table 4, no differences between male and female dogs were found (*p* > 0.05). In group 1, the only difference found between sterilized and unsterilized dogs was in walking the dog at least once a day (Z = −2.479; *p* = 0.013), but this difference was not significant in group 2. Within group 1, sterilized female dogs were walked more frequently than were unsterilized female dogs (Z = 2.454; *p* = 0.014), and no differences between sterilized and unsterilized male dogs were found.

The different behaviors evaluated by the C-BARQ were considered indicators of benefits for the dog. Significant differences in most behaviors were found, with fewer aggressive behaviors in any modality, less fear, and higher trainability scores in dogs with greater compatibility in activity preferences with their owners. The differences in chasing, separation anxiety, excitability and attention-seeking behaviors or energy level were not significant (Table 5).

Within each group, with regard to the C-BARQ scores, some differences between male and female dogs were found in group 1 but not in group 2. These differences were in dog-directed fear (Z = −2.219; *p* = 0.026), chasing (Z = −2.203; *p* = 0.031) and stranger-directed fear (Z = −2.160; *p* = 0.031), with female dogs having higher scores.

Between sterilized and unsterilized dogs, no differences in group 2 were found. Within group 1, sterilized dogs showed higher scores in trainability (Z = −2.846; *p* = 0.004) and excitability (Z = −2.180; *p* = 0.029).

Within each group and within the same sex, between sterilized and unsterilized dogs, no differences in the males of group 1 were found. Trainability was higher in sterilized females (Z = −3.139: *p* = 0.002). All other differences were not significant.

The sterilized males of group 2 showed higher scores in dog rivalry (Z = −2.117; *p* = 0.034). Unsterilized males showed higher scores in touch sensitivity (Z = −2.524; *p* = 0.012) and in attachment/attention-seeking (Z = −2.115; *p* = 0.034). Additionally, the sterilized females of group 2 showed higher scores in dog-directed fear (Z = −2.178; *p* = 0.029). All other differences were not significant.

## 4. Discussion

When selecting a companion animal, compatibility in human-dog dyads is an aspect that should be considered. Ideally, owners will choose a dog based on their personal activity levels; thus, active people will select dogs that can be their companions on walks, while sedentary people will select less active breeds or companion animals that do not require as much exercise [6].

Considering that relationships between humans and animals have consequences for both parties that range from the physical to the psychological [1], the purpose of this study was to compare some indicators of the benefits of human-dog interaction between people with and without compatible activity preferences with their dogs. The groups were equivalent in terms of the characteristics evaluated, with the exception of dog age and years living with the family; the dogs in the group with lower compatibility were older dogs and had lived with the family longer, although the average difference was only 1 year. According to the measures of central tendency, the dogs in both groups were young adults; thus, a comparative analysis by age was not considered.

Previous studies have found that dog age is related positively to some aggressive behaviors and negatively to separation-related behaviors and energy level [4]. For this reason, future studies should compare the behaviors evaluated by the C-BARQ between dogs of different developmental stages: puppies, adolescents, adults, and seniors.

Regarding the indicators of benefits for humans, in agreement with previous studies, it was found that the more compatible group had lower perceived stress scores. Previously, it was found that dog owners had lower stress levels than nonowners [23], and the positive effect of companion animals on stress responses has also been reported [24].

Regarding subjective happiness, in previous studies, this variable showed no differences between dog owners and nonowners [23]. Similarly, another study reported a weak correlation between subjective happiness and the human–dog relationship and found no direct effect based on structural equation modeling [15]. That study concluded that the benefits of human–animal interaction are mediated by the quality of the relationship with the dog, which coincides with the present results, considering that the more compatible individuals had a higher-quality relationship with their dog and higher happiness scores.

The third indicator of benefits for humans was the number of doctor visits in the last year. No significant differences were found in this indicator between people with and without compatibility with their dog’s activity preferences. This indicator has been used in previous studies [1,25], including epidemiological and longitudinal studies revealing that people who lived with companion animals had fewer doctor visits than similar people without companion animals [1] and a longitudinal study with data from Germany and Australia showing that owners of companion animals had 15% fewer doctor visits per year compared to nonowners [25]. Considering the above, it is concluded that compatibility does not explain the doctor visits indicator. However, this indicator has its limitations because it is self-reported; thus, in future studies, it is necessary to use other indicators of physical health.

The dog routine indicators show that owners who are more compatible also have a better routine with regard to engaging in daily play and daily walks and feeding the dog at fixed times. These aspects can provide benefits for both the human and the dog. Exercise and feeding management are associated with obesity in dogs [26], and play is a benchmark for behavioral compatibility in activity preferences [3]. Previously, it has been found that people with dogs take longer [12] and more frequent walks than do nonowners and that in older adults, walking the dog has been associated with greater improvement in physical health than walking with other humans [27].

In the majority of the behaviors evaluated by the C-BARQ, significant differences between the groups were found. The intention was not to attribute causality between the compatibility of activity preferences and dog behavior; instead, the hypothesis was that the differences would arise because the more compatible dyads had a better relationship. Thus, the humans would perceive the behaviors of their dogs as less bothersome. In addition, it has previously been reported that when humans spend short periods of time at home, there is a greater risk of behavioral problems in their dogs [28]. Therefore, when humans and their dogs share more activities and time together, the dogs will focus their energy on behaviors that are more acceptable to their humans.

The frequency of instinctive behaviors, such as chasing, or of behaviors and characteristics that reflect the dog’s temperament, such as excitability, attention-seeking and energy level, were equivalent between the groups. There were also no differences in separation-related problems, a result that coincides with previous research reporting equivalences in compatibility in activity preferences between groups with and without separation-related problems [29].

The results of the different subscales of the C-BARQ may be explained by the fact that in the more compatible dyads, the needs of the dogs were better met, a finding that is consistent with previous reports on the influence of owner activity levels on the exercise needs of dogs [6].

Considering a recent review by Scandurra et al. [30], who reported differences between sexes in dogs in some variables, it is possible that the differences found in this study can be explained by the sex of the dogs or their sterilization status. However, in the variables for which differences were found, these differences are not consistent in both groups; thus, compatibility can be an important variable to consider, regardless of the sex of the dog.

It is possible that people who have sterilized female dogs feel more comfortable walking them; thus, they walk them more frequently. However, this difference was found only in group 1, consisting of individuals with less than 50% compatibility.

Also, in group 1, female dogs had higher scores in dog-directed fear, chasing and stranger-directed fear, which coincides with results reported in the review by Scandurra et al. [30].

Sterilized dogs showed higher scores in trainability and excitability, but these behaviors did not show differences between sexes, although Scandurra et al. [30] mentioned that females tended to be more excitable than males. A more detailed analysis showed that trainability was higher in sterilized females within group 1.

Within group 2, sterilized males showed higher scores in dog rivalry. Unsterilized males showed higher scores in touch sensitivity and attachment/attention-seeking. Sterilized females showed higher scores in dog-directed fear only within group 2.

Based on sex or sterilization status, no differences in behaviors related to aggressiveness were found, which is not consistent with the findings of the review by Scandurra et al. [30], who mentioned several studies that reported that ovariectomy results in increased aggressive behaviors in females. These inconclusive results motivate the suggestion that future research on human–animal interaction can focus on the differences between males and females and on the sterilization status of dogs.

An important aspect to consider is that the dog-owner compatibility index of activity preferences evaluates the behavioral component of compatibility, referring to the shared enjoyment of daily activities such as walking and interacting with others [5]. People and dogs with active lifestyles seem to complement each other [31]; however, future research should evaluate the other components of compatibility: the physical and the psychological.

Continuing this line of research is relevant because companion animals play a significant role in today’s society [7] and are considered family members by approximately 90% of owners [1]. However, it should also be considered that the results of available studies disagree, perhaps due to differences in the variables included, the methodological designs and the lack of control of extraneous variables [7]. This last element was controlled in this study through the inclusion of groups that were equivalent in terms of various variables, although limitations related to sample size and type of sampling are acknowledged.

Some limitations should be mentioned. No objective measures were used; thus, the results are based on owner perceptions. The sample size and sampling method are additional limitations. It is important to consider that in Mexico, there are 23 million dogs and cats, and between 57 and 70 out of every 100 households have pets, with dogs being the favorite animal, according to the National Institute of Statistics and Geography (Inegi) [32]. This study represents a contribution to this line of research; however, its results cannot be considered representative of the country.

## 5. Conclusions

Compatibility in preferred activities helps explain the benefits of human–animal interaction.

## Figures and Tables

**Table 1:** Characteristics of the participants and their dogs (numerical variables).

Variable	G1 Md	G1 M	G1 SD	G1 IQR	G2 Md	G2 M	G2 SD	G2 IQR	Mann–Whitney U
Age of human	30.0	31.0	7.1	11.0	31.5	31.9	6.2	9.0	Z = −1.223; *p* = 0.221
Years of marriage or living with a partner	3.0	4.5	5.5	6.0	3.0	4.5	4.3	5.0	Z = −0.876; *p* = 0.381
Dogs in the home	2.0	2.1	1.5	2.0	2.0	2.0	1.3	1.0	Z = −0.189; *p* = 0.850
Age of the dog	3.0	4.4	3.4	4.3	3.0	3.4	2.8	4.0	Z = −2.274; *p* = 0.023
Years with the dog	3.0	4.0	3.2	4.0	2.1	3.1	2.8	3.7	Z = −2.610; *p* = 0.009

G1 (*n* = 90): group 1, with compatibility from 0 to 50%. G2 (*n* = 110): group 2, with 100% compatibility. Md: Median; M: Mean; SD: Standard deviation; IQR: interquartile range.

**Table 2:** Characteristics of the participants and their dogs (categorical variables).

Variable	Group 1 Frequency	Group 1 Percentage	Group 2 Frequency	Group 2 Percentage	Chi-Square Test
Sex:					X^2^ = 1.191; *p* = 0.275
Female	78	86.7	89	80.9	
Male	12	13.3	21	19.1	
Marital status:					X^2^ = 4.688; *p* = 0.321
Single	49	54.5	48	43.6	
Married or with partner	37	41.1	60	54.6	
Separated or divorced	4	4.4	2	1.8	
Has children (yes)	18	20	17	15.5	X^2^ = 0.708; *p* = 0.400
Works (yes)	66	73.3	88	80.0	X^2^ = 1.242; *p* = 0.265
Considers the dog a member of the family (yes)	77	85.6	103	93.6	X^2^ = 3.591; *p* = 0.058
Sex of the dog: Male	41	45.6	56	50.9	X^2^ = 0.568; *p* = 0.451
Dog sterilized (no)	62	68.9	65	59.1	X^2^ = 2.050; *p* = 0.152
Size of the dog:					X^2^ = 6.465; *p* = 0.167
Miniature, 3 to 5 kg	23	25.6	14	12.7	
Small, 5 to 12 kg	29	32.2	39	35.5	
Medium, 12 to 25 kg	19	21.1	29	26.4	
Large, 25 to 40 kg	18	20.0	24	21.8	
Giant, >40 kg	1	1.1	4	3.6	

**Table 3:** Differences between groups in the indicators of benefits for humans.

Variable	G1 Md	G1 M	G1 SD	G1 IQR	G2 Md	G2 M	G2 SD	G2 IQR	Mann-Whitney U
Subjective happiness (mean)	5.3	5.1	1.2	1.7	5.5	5.5	0.8	1.2	Z = −2.831; *p* = 0.021
Perceived stress	22.0	21.7	8.1	10.0	19.0	18.5	6.9	9.0	Z = −2.831; *p* = 0.005
Number of doctor visits in the last year	2.0	3.1	4.0	3.0	2.0	2.1	2.3	2.0	Z = −1.729; *p* = 0.084

G1 (*n* = 90): group 1, with compatibility from 0 to 50%. G2 (*n* = 100): group 2, with 100% compatibility. Md: median; M: mean; SD: standard deviation; IQR: interquartile range.

**Table 4:** Differences between groups with regard to their routines with their dog.

Variable	G1 Md	G1 M	G1 SD	G1 IQR	G2 Md	G2 M	G2 SD	G2 IQR	Mann-Whitney U
Walking the dog at least once a day	2.0	2.4	1.0	1.0	3.0	3.2	0.9	1.0	Z = −5.851; *p* = 0.001
Feeding at fixed times	4.0	3.0	1.2	2.0	4.0	3.6	0.8	0.0	Z = −3.924; *p* = 0.001
Food bowl available all day long	2.0	2.5	1.4	3.0	1.0	2.0	1.3	2.0	Z = −2.153; *p* = 0.031
Frequency of play with the dog	4.0	3.7	1.3	2.0	4.0	4.3	0.8	1.0	Z = −3.278; *p* = 0.001

G1 (*n* = 90): group 1, with compatibility from 0 to 50%. G2 (*n* = 110): group 2, with 100% compatibility. Md: median; M: mean; SD: standard deviation; IQR: interquartile range.

**Table 5:** Differences between groups with regard to the C-BARQ scores.

Variable	G1 Md	G1 M	G1 SD	G1 IQR	G2 Md	G2 M	G2 SD	G2 IQR	Mann-Whitney U
Stranger-directed aggression	3.1	3.2	0.8	1.1	2.7	2.8	0.7	1.0	Z = −3.675; *p* = 0.001
Owner-directed aggression	0.2	0.4	0.6	0.6	0.0	0.2	0.4	0.2	Z = −3.365; *p* = 0.001
Dog-directed aggression	1.2	1.5	1.1	1.8	0.5	0.8	0.9	1.3	Z = −4.111; *p* = 0.001
Dog-directed fear	0.7	1.1	1.1	1.7	0.5	0.6	0.7	1.0	Z = −2.800; *p* = 0.005
Dog rivalry (familiar dog aggression)	0.5	0.9	1.0	1.5	0.0	0.4	0.8	0.5	Z = −3.569; *p* = 0.001
Trainability	2.1	2.0	0.8	1.0	2.5	2.5	0.7	1.0	Z = −4.007; *p* = 0.001
Chasing	1.0	1.2	0.9	1.3	0.8	1.1	1.0	1.5	Z = −1.174; *p* = 0.240
Stranger-directed fear	0.6	0.9	1.0	1.5	0.0	0.5	0.8	0.8	Z = −2.769; *p* = 0.006
Nonsocial fear	1.0	1.2	0.8	1.0	0.7	0.8	0.7	1.0	Z = −3.280; *p* = 0.001
Separation-related problems	0.9	0.9	0.7	1.1	0.6	0.7	0.6	0.7	Z = −1.121; *p* = 0.262
Touch sensitivity	0.9	1.1	0.9	1.2	0.5	0.7	0.8	1.0	Z = −3.789; *p* = 0.001
Excitability	2.2	2.2	0.9	1.0	2.0	2.1	0.9	1.0	Z = −0.881; *p* = 0.378
Attachment/attention-seeking	2.2	2.3	0.9	1.0	2.3	2.3	0.8	1.2	Z = −0.284; *p* = 0.776
Energy	1.5	1.3	0.9	1.5	1.5	1.5	1.0	1.0	Z = −1.601; *p* = 0.109

G1 (*n* = 90): group 1, with compatibility from 0 to 50%. G2 (*n* = 110): group 2, with 100% compatibility. Md: median; M: mean; SD: standard deviation; IQR: interquartile range.
